# Greater Adolescent Cognitive Ability Is Linked to Lower Risk of Cognitive Impairment in Later Life

**DOI:** 10.1093/geroni/igab046.1853

**Published:** 2021-12-17

**Authors:** Tara Gruenewald, Catalina Zavala, Molli Grossman, Thalida Arpawong, Jennifer Manly, Susan Lapham, Margaret Gatz, Carol Prescott

**Affiliations:** 1 Chapman University, Orange, California, United States; 2 University of Southern California, Los Angeles, California, United States; 3 Columbia University, Columbia University, New York, United States; 4 American Institutes for Research, American Institutes for Research, Virginia, United States

## Abstract

There have been few investigations of the role that adolescent cognitive ability plays in predicting later-life cognitive impairment, and the mechanisms, such greater life course educational exposure, that might underlie these connections. This knowledge gap is due, in part, to a lack of cohorts with early-life cognitive assessment who are followed to later adulthood. We capitalized on data from the 1960 Project Talent (PT) high school cohort (n>360,000) and two recent follow-ups, the Project Talent Twin & Sibling (PTTS; n=2,491 in 2014) Study and the Project Talent Aging Study (PTAS; n=6,421 in 2018), to examine these potential links. In 1960, ability was assessed in multiple cognitive domains (e.g., general aptitude, quantitative, reasoning). Participants/proxies reporting 2 or more symptoms of cognitive impairment in 2018 on the AD8 Dementia Screener were classified as having a positive screen. Binary logistic generalized estimating equations with race, sex, and adolescent family SES covariates, indicated that in multiple cognitive domains, higher ability in adolescence predicted lower odds of a positive AD8 screen in later life (ORs of 0.80 - 0.85). The effects were only slightly attenuated with inclusion of life course educational attainment. Sibling models found a similar pattern of associations and effect sizes, indicating that the association is not attributable to shared family and genetic background. These findings indicate that higher cognitive ability as indicated by better performance in multiple cognitive domains in adolescence may be protective against cognitive impairment five decades later and life course educational attainment only partially mediates this association.

